# Coated Tongues

**Published:** 1884-11

**Authors:** 


### COATED TONGUES.

AMONG the various substances which have been found on the human tongue, as shown by the microscope, are the following: Fibers of wool, linen, and cotton; fibers of spiral vessels; fibers of muscle, in one case eight hours after eating; starch grains; cheese mould; portions of potato skin; scales, moths, etc.; hairs from legs of bees; hairs from legs of spiders; pollen of various flowers; stamens of various flowers; hairs of cats, quite common; hairs of mouse once only; hairs from various leaves; wing of mosquito once; fragments of the leaves of tobacco, of chamomile flowers, etc.

**An Electric Railway**.—The longest electric railway in the world is that between Portrush and the Giant's Causeway, in Ireland, a distance of six miles. Its cost was $225,000. The force to work it is generated by a waterfall in the Bush River, which has a head of 24 feet and is equal to about 90-horsepower. There is no doubt as to the success of this venture, as the power costs nothing. This road is a tramway, but the other electric railways in Berlin and elsewhere are elevated roads. A New York inventor is about to introduce an electrical engine of 2-horsepower for propelling cabs and carts through streets, and yachts and other small boats through water. Electrical machines take very little room, make no noise, require no coal, and no doubt will at some time in the future supersede horseflesh in propelling vehicles of all kinds through the streets of cities and over country roads.

Said a sleeping-car porter to me the other day, "Ladies are four times as much trouble as men when travelling." "No doubt," said I; "but then they are four times as nice—which balances the account." As this is the most gallant thing I ever said, the ladies will observe the propriety of sending in their tokens of affection and appreciation before I take it back.

"My dear, look down below," said a grandiose, as he stood on the bridge with his wife, and gazed at a tug hauling a long line of barges. "Such is life—the tug is like a man, working and toiling, while the barges, like women, are—" "I know," interrupted Mrs. G., acridly; "the tug does all the blowing, and the barges bear all the burden"

**Managing a Stove.**—What every one can do, few will do. The greenest "Biddy" thinks she can manage a stove, and resents the instruction of the mistress, who may be as ignorant of this simple art as her servant. The following directions would, if heeded, save both stove and fuel, besides keeping the fire always in working order. Miss Parloa, in a recent lecture in New York, said one of the most frequent mistakes people make is in putting on too much coal.

Never have the coal come above the lining of the stove. It is a waste of fuel, and the fire will not be so bright and clear, because the draught will not be so good.

When not using the fire, keep the dampers closed; it will be ready when needed; then open the draughts.

For cooking, either on top of the stove or in the oven, no matter how hot the fire desired, having the coal come nearly to the top of the lining, the fire ought to last four hours without new coal or poking.

The top of the stove may be red-hot, and the coal piled upon the lids, and yet the oven will not bake. It is because there is too much coal, and the draught is stopped by it. The practice of having the top of the stove or range red-hot will soon destroy it.—*Youth's Companion.*

